# The World’s Great Religions and Their Neglected Tropical Diseases

**DOI:** 10.1371/journal.pntd.0004544

**Published:** 2016-07-28

**Authors:** Peter J. Hotez

**Affiliations:** 1 Sabin Vaccine Institute and Texas Children’s Hospital Center for Vaccine Development, National School of Tropical Medicine, Baylor College of Medicine, Houston, Texas, United States of America; 2 James A Baker III Institute for Public Policy, Rice University, Houston, Texas, United States of America; 3 Department of Biology, Baylor University, Waco, Texas, United States of America; 4 Scowcroft Institute for International Affairs, The Bush School of Government and Public Service, Texas A&M University, College Station, Texas, United States of America; Molecular Biology Unit (MBU), INDIA

New information based on data released by the World Health Organization (WHO) indicates that practically everyone infected with a major neglected tropical disease (NTD) lives in a Christian-, Muslim-, or Hindu-majority nation. The finding has implications for engaging religious leaders in NTD control and elimination activities.

**Fig 1:**
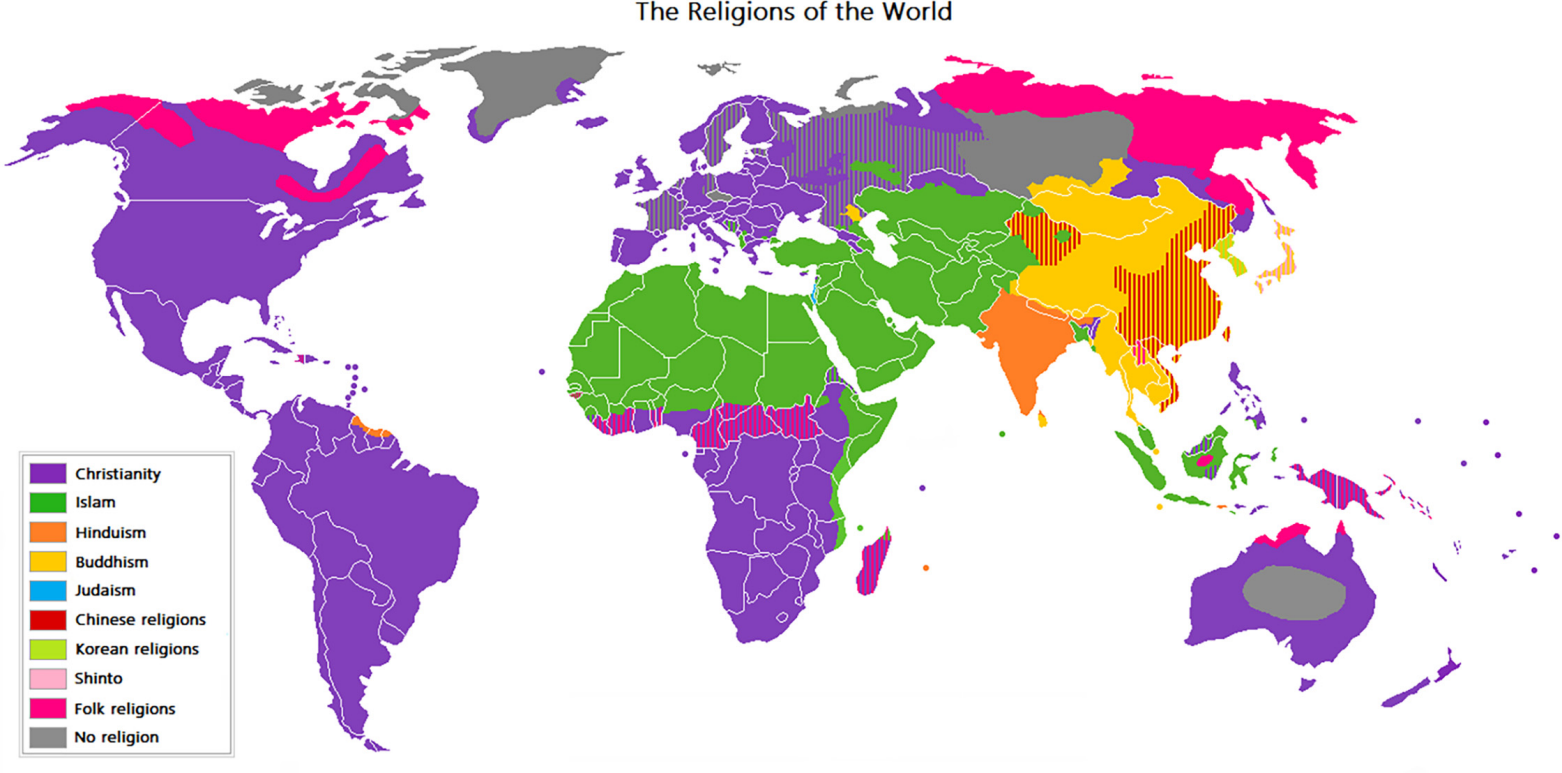
The religions of the world, mapped by distribution. https://en.wikipedia.org/wiki/Major_religious_groups#/media/File:Religion_distribution.png

Today, of the estimated 7.4 billion (thousand million)_people living [1], approximately one-half to two-thirds are linked to the three largest religions: Christianity (2.0–2.2 billion people), Islam (1.2–1.6 billion), and Hinduism (0.8–1.0 billion) [2,3]. As shown in Fig 1, the world’s religions are not evenly distributed. The Muslim-majority countries that comprise the Organization of Islamic Cooperation (OIC) are found in the Middle East and North Africa region, extending down to the African Sahel, as well as in Southeast Asia. Christian-majority countries comprise those in the Western Hemisphere, Europe, central and southern Africa, Philippines, and Australia. The Hindu-majority countries are composed of India, Nepal, and Mauritius.

In previous papers published in *PLOS Neglected Tropical Diseases*, we have pointed out the disproportionate impact of NTDs on Muslim-majority countries [4,5], Christian-majority countries [6,7], and in India [8,9].

With respect to the OIC nations, new studies by the Pew Research Center find that Muslims represent the fastest growing religious group and possibly the only one growing faster than the global population [2]. Pew further projects that the number of adherents to Islam will become roughly equal to Christians by the year 2050 [2]. We have found that poverty and NTDs disproportionately affect the Muslim-majority nations [4,5]. For instance, based on WHO Parasite Chemotherapy and Transmission Control (PCT) databases from 2012–2013 [10–12], it was determined that while populations living in the 30 largest Muslim-majority nations (representing 90% of people living in OIC countries) comprise only about 20% of the global population, they account for 50% and almost 40% of the world’s school-aged children requiring preventive chemotherapy treatments for schistosomiasis and intestinal helminth infections, respectively, as well as one-third of the population that requires preventive chemotherapy for lymphatic filariasis (LF) [5]. Some of the hardest-hit OIC countries with respect to NTDs include those in Sahelian Africa, which have the highest worm indices of impaired development [5], Indonesia and Bangladesh in Southeast Asia, and some of the poorest countries in the Middle East and North Africa (MENA). The worm index is a composite that includes school-aged children requiring mass treatment for their schistosomiasis and intestinal helminth infections as well as populations needing mass drug administration (MDA) for LF divided by the estimated population [5]. The worm index is inversely related to the human development index (HDI). Beyond poverty and low HDI, conflicts in the Islamic State (IS)-occupied territories in Syria, Iraq, Libya, as well as in Yemen are also contributing to the further emergence of NTDs such as leishmaniasis [13].

Similarly, some key and highly populated Christian-majority populations are profoundly affected by NTDs, partciularly since for the first time more Christians are now living in developing regions of Africa, Asia, and Latin America than in Europe and North America [7]. Table 1 shows an updated analysis of NTDs in the largest Christian-majority nations in the Latin America and Caribbean region, Asia (Philippines), and Africa (DR Congo, Ethiopia, South Africa, Kenya, Angola, Ghana, Malawi, Zambia, and Zimbabwe), each with a population greater than 10 million [10–12,14–16]. Based on WHO PCT databases, these countries and regions account for 40% of the world’s school-aged children requiring MDA for schistosomiasis, almost one-quarter of those requiring treatment for intestinal helminth infections, and 13% of the poulation needing LF preventive chemotherapy. Even though 2014 PCT data are now available, for this analysis, the 2012–2013 data were used to keep it consistent with the previous analysis for OIC nations.

**Table 1:** NTDs in Christian-majority developing countries and regions.

Country	School-aged children requiring treatment for intestinal helminth infections in 2013 [10]	School-aged children requiring treatment for schistosomiasis in 2013 [11]	Populations requiring preventive chemotherapy for LF in 2013 [12]
Latin America and Caribbean region	33,282,146 ^a^	1,564,885^b^	12,048,009^c^
Philippines	22,184,699	508,621	19,541,323
DR Congo	18,449,259	10,167,441	49,140,000
Ethiopia	25,897,255	11,979,190	30,000,000
South Africa	2,637,454	2,476,276	0
Kenya	11,673,615	5,810,844	3,421,741
Angola	6,172,782	2,840,832	12,090,000
Ghana	364,776	3,175,687	10,237,354
Malawi	4,557,597	3,006,186	14,989,401
Zambia	4,111,764	2,317,543	8,780,000
Zimbabwe	2,136,454	1,541,427	6,000,000
Total	131,467,801	45,388,932	166,247,828
Total globally	609.5 million in 2012 [14]	114.3 million in 2012 [15]	1,241.9 million reported in 2013 [16]
% of global in Christian-majority nations	22%	40%	13%

^a^Number obtained from WHO (2015) Soil-transmitted helminthiases: number of children treated in 2013. Weekly Epidemiol Rec 90: 89–96

^b^Number obtained from WHO PCT data for the four schistosomiasis-endemic countries in 2013: Brazil, Dominican Republic, Suriname, and Venezuela

^c^Number obtained from WHO PCT data for the four LF-endemic countries in 2013: Brazil, Dominican Republic, Guyana, and Haiti.

Table 2 shows a similar analysis for the three Hindu-majority nations of India, Nepal, and Mauritius. It demonstrates that together these three countries account for 41% of the world’s population requiring preventive chemotherapy for LF and more than one-quarter of the school-aged children requiring mass drug administration for their intestinal helminth infections. Schistosomiasis is not endemic to South Asia.

**Table 2:** NTDs in Hindu-majority developing countries: India, Nepal, and Mauritius.

Country	School-aged children requiring treatment for intestinal helminth infections in 2013 [10]	School-aged children requiring preventive chemotherapy for treatments for schistosomiasis in 2013 [11]	Populations requiring preventive chemotherapy for LF in 2013 [12]
India	157,498,685	0	489,133,952
Nepal	6,724,600	0	21,852,201
Mauritius	15,659	0	0
Total	164,238,944	0	510,986,153
Total globally	609.5 million in 2012 [14]	114.3 million in 2012 [15]	1,241.9 million reported in 2013 [16]
% global in Hindu-majority nations	27%	0	41%

A summary of the three major helminth infections—intestinal helminth infections, schistosomiasis, and LF—is shown in Table 3. It shows that the OIC and Christian-majority countries account for 90% of the world’s school-aged children requiring MDA for schistosomiasis. Adding in the three Hindu-majority countries, the majority of the nations of the world’s three largest religions also account for almost 90% of the children requiring MDA for intestinal helminth infections and populations needing treatment for LF. A previous analysis found that Christian-majority countries also account for almost all of the world’s cases of Chagas disease and human African trypanosomiasis [7].

**Table 3:** Percentage of NTDs in OIC countries, Christian-majority developing countries and regions, and the three Hindu-majority nations.

Religious-affiliated country or region	School-aged children or children requiring treatment for intestinal helminth infections	School-aged children requiring preventive chemotherapy for treatments for schistosomiasis	Populations requiring preventive chemotherapy for LF	Total helminths
OIC Countries	37%	50%	33%	35%
Christian-majority developing countries/regions	22%	40%	13%	17%
Hindu-majority countries	27%	0	41%	34%
Total	86%	90%	87%	86%

A key implication from these findings is that helminth infections represent critical factors in holding back the health and economic development of Christian-, Muslim-, and Hindu-majority developing nations, while Chagas disease and HAT are also devastating Christian-majority countries in Latin America and Africa, respectively. It is likely that a similar analysis might also hold for additional NTDs such as leishmaniasis and leprosy. These diseases are important causes of poverty because of their deleterious impact on pregnancy and on girls and women in general, child development, and adult worker productivity.

Linking NTDs to religion has potential importance because it invites prominent international religious leaders to have a greater role in advocating for and supporting NTD control [9]. For instance, for the OIC nations, the Islamic Development Bank, and some of the wealthier nations of the Gulf Cooperation Council could look at opportunities to contribute to MDA as well as supporting research and development (R&D) for new technologies. Through the United States Science Envoy program, we recently embarked on a cooperative R&D agreement between the Sabin Vaccine Institute and King Saud University for NTD vaccine development. Similarly, Pope Francis has been a staunch advocate for the poor and could add NTDs into the portfolio of activities for the Papacy, while leaders in India and Nepal can expand their existing commitments to NTDs, especially for intestinal helminth infections, LF, leishmaniasis, and other conditions. At the local level, religious leaders in churches, mosques, and temples could have important roles in raising awareness about NTDs and their health impact and could even promote indigenous control and elimination efforts.

Finally, there remains the interfaith opportunity to bring these three great religions together in order to cooperate on reducing the global burden of NTDs. NTD control and elimination represents one of the most effective and cost-efficient means to reduce poverty and relieve global suffering.
